# The development of a decision aid to support Hodgkin lymphoma survivors considering lung cancer screening

**DOI:** 10.1186/s12911-022-01768-y

**Published:** 2022-02-01

**Authors:** Rachel Broadbent, Tania Seale, Christopher J. Armitage, Kim Linton

**Affiliations:** 1 Division of Cancer Sciences, School of Medical Sciences, Faculty of Biology, Medicine and Health, University of Manchester, Manchester, UK; 2 NIHR Greater Manchester Patient Safety Translational Research Centre, University of Manchester, Manchester, UK; 3 The Christie NHS Foundation Trust, Manchester, UK; 4 Division of Psychology and Mental Health, Manchester Centre for Health Psychology, University of Manchester, Manchester, UK; 5 Manchester Academic Health Science Centre, Manchester University NHS Foundation Trust, Manchester, UK; 6 Manchester Cancer Research Centre, Wilmslow Road, Manchester, M20 4QL UK

**Keywords:** Hodgkin lymphoma, Lung cancer screening, Decision aid

## Abstract

**Background:**

Decisions aids (DA) can support patients to make informed decisions about screening tests. This study describes the development and initial evaluation of a lung cancer screening (LCS) DA targeted towards survivors of Hodgkin lymphoma (HL).

**Methods:**

A prototype decision aid booklet was developed and subsequently reviewed by a steering group who provided feedback. Revisions were made to produce the DA tested in this study. HL survivors were recruited to an online survey and/or focus groups. Lymphoma practitioners were invited to an interview study. In the online survey, decisional conflict scales and knowledge scales were completed before and after accessing the DA. The focus groups and interviews explored acceptability and comprehensibility and the decisional needs of stakeholders. Focus groups and interviews were audio recorded. The framework method was used to analyse qualitative data.

**Results:**

38 HL survivors completed the online survey. Following exposure to the DA, knowledge of LCS and risk factors and decisional conflict scores (total score and subscale scores) improved significantly. 11 HL survivors took part in two focus groups (n = 5 and n = 6) and 11 practitioners were interviewed. Focus group and interview results: The language, format and length were considered acceptable. Both groups felt the DA was balanced and presented a choice. Icon arrays were felt to aid comprehension of absolute risk values and for some survivors, they reduced affective risk perceptions. Among survivors, the impact of radiation risk on decision making varied according to gender and screening interval, whilst practitioners did not anticipate it to be a major concern for patients. Both groups expressed that a screening offer could mitigate anxiety about lung cancer risk. As anticipated by practitioners, survivors expressed a desire to seek advice from their clinical team. Practitioners thought the DA would meet their informational needs regarding LCS  when supporting survivors.

**Conclusions:**

The DA is considered acceptable by HL survivors and practitioners. The DA reduces decisional conflict and improves knowledge in HL survivors, suggesting that it would support HL survivors to make informed decisions when considering LCS in a future clinical trial.

**Supplementary Information:**

The online version contains supplementary material available at 10.1186/s12911-022-01768-y.

## Background

People invited to undergo cancer screening must be provided with information to support informed decision making about participation, in keeping with the General Medical Council guidelines on decision making and consent [1]. In the UK, guidance issued by NHS Cancer Screening Programmes stipulates that screening programmes should provide patients with educational materials covering the purpose of the investigation, the risks, benefits and burdens of the screening test and the likelihood of the test outcomes [2].

Decision aids are evidence-based tools which should support patients in their decision making when facing healthcare options and help patients to make explicit decisions in accordance with their personal values [3]. An updated Cochrane systematic review examined the use of decision aids in people facing healthcare or screening decisions and found that compared to usual care, decision aids improve knowledge and accuracy of risk perception, increase value-based decision making and reduce decisional conflict related to feeling uninformed, thus improving the quality of decision making [3]. A number of decision aids have been developed to support patients making decisions about cancer screening, including ever smokers considering lung cancer screening [4–6] and those with low literacy levels considering bowel cancer screening [7, 8].

Hodgkin lymphoma (HL) is a malignancy of clonal B-cells which mainly affects young adults and the elderly [9]. Due to the carcinogenic effects of thoracic radiotherapy and chemotherapy, survivors of HL are at excess risk of developing lung cancer (30-year cumulative incidence 6.4%) [10, 11]. Lung cancer screening has been implemented for ever smokers over the age of 55 [12, 13], but most HL survivors will not be eligible for screening as the majority are non-smokers [14]. Clinical trials of lung cancer screening for Hodgkin lymphoma survivors are underway [15, 16], but to our knowledge, educational materials to support decision making have not been developed. Existing lung cancer screening education materials are targeted towards ever smokers and are not appropriate for HL survivors as they do not address treatment related lung cancer risk. Prior research has found that HL survivors have a low perceived risk of lung cancer due to a lack of awareness of the risks associated with cancer treatment [17]. There is a need to develop educational materials targeted towards HL survivors considering lung cancer screening to use in future trials and screening programmes. To address this, we have developed a decision aid for use in a future trial of lung cancer screening. This paper describes the design and development process.

## Methods

### The aim and scope of the decision aid

Our aim was to develop a decision aid for use in a future trial of lung cancer screening using low-dose CT scans in at risk HL survivors. The decision aid is intended to support HL survivors who are deciding whether to undergo lung cancer screening as part of the study.

### Content development

The International Patient Decision Aids Standards instrument (IPDASi) was used to guide content development [18]. Published literature informed the manner in which risk information is presented in the decision aid. Evidence has shown that presenting absolute risk values improves accuracy of risk perceptions compared to relative risk values [19–22] and there is a consensus in the literature that the absolute risk format is the optimal method for presenting risk data [20, 21]. Therefore, absolute risk values are presented in the decision where possible, avoiding the use of relative risk, or numbers needed to screen. Where absolute risk information was not published, the Winton Centre for Risk and Evidence Communication Real Risk-Make Sense of Your Stats website [22] was used to calculate absolute risk by extracting data from published literature. Absolute risk values are accompanied throughout the decision aid by visual aids in the form of icon arrays, which have been shown to improve accuracy and comprehension of risk perception [19, 23]. There are instances where risk is presented qualitatively in the decision aid: firstly, to describe the greater likelihood of lung cancer in HL survivors who have smoked and secondly in HL survivors with a family history of lung cancer. In these specific examples, published data did not provide absolute risk values (or the raw data required to calculate these independently). In the example of smoking history, absolute risk values calculated from the sole paper providing raw data was misleading in that it suggested that HL survivors who are never smokers do not develop lung cancer. Since the literature suggests that most HL survivors who develop lung cancer have a history of smoking, we used the statement “most people who get lung cancer after Hodgkin lymphoma have smoked” in the decision aid.

**Fig. 1:**
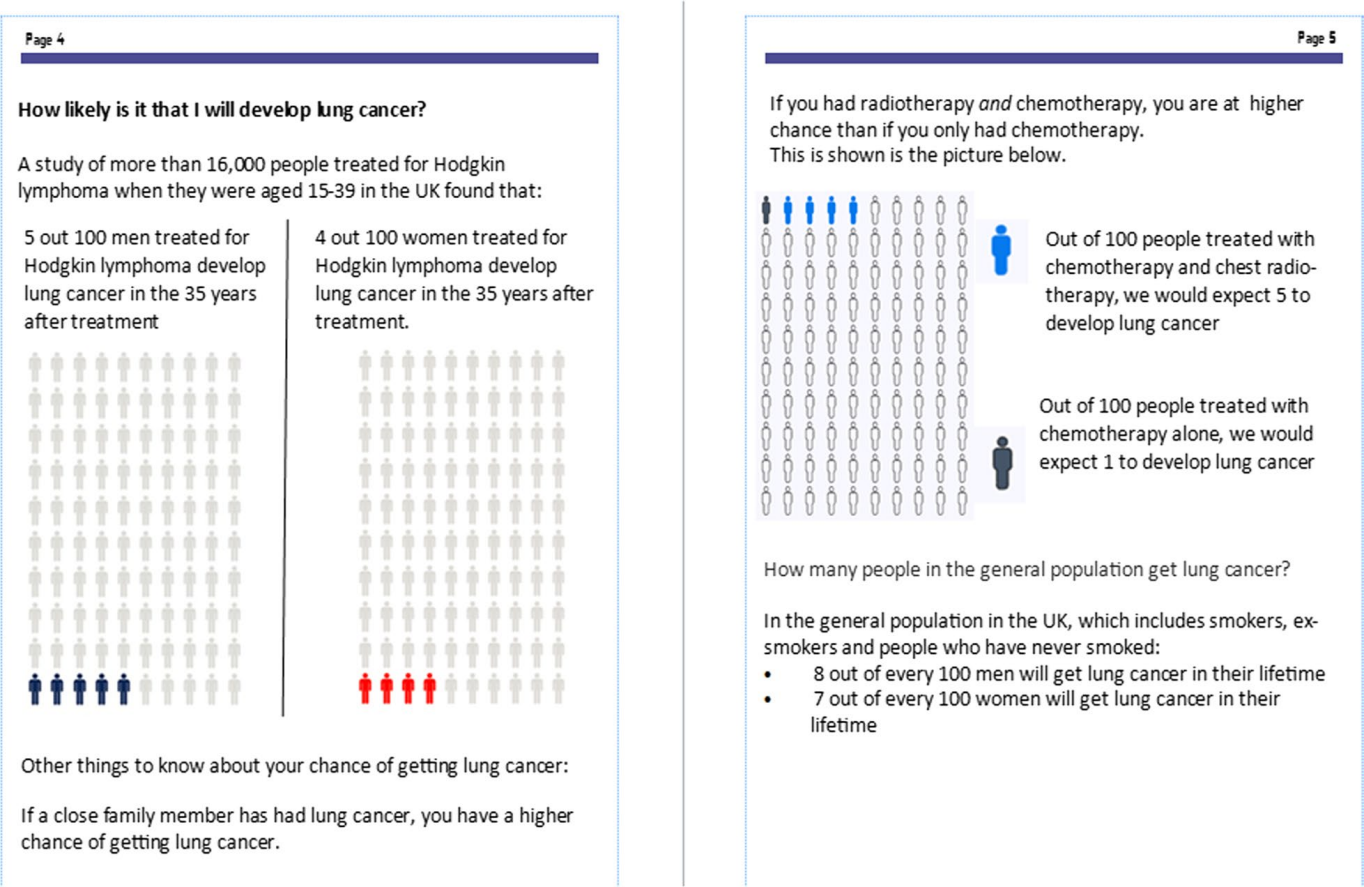
Screenshot of the pages in the decision aid containing icon arrays demonstrating absolute risk of lung cancer according to gender and treatment for HL

There is strong evidence from a Cochrane systematic review [24] that personalised risk communication promotes informed uptake of screening tests and increases knowledge. For this reason, there are two icon arrays in the decision aid, which demonstrate absolute lung cancer risk in men and women and absolute lung cancer risk according to whether the survivor was treated with chemotherapy alone or chemotherapy and radiotherapy. The pages of the decision aid containing these icon arrays can be seen in Fig. 1. In the absence of a lung cancer risk calculator for this population, it was not possible to provide individualised risk scores.

Published literature also informed the information in the decision aid regarding risk factors for developing lung cancer after treatment for Hodgkin lymphoma [10, 11, 25, 26], cumulative incidences and absolute risk levels [25, 27]. Information about the lung cancer screening test was informed by publicly available information and an online lung cancer screening decision tool [28, 29] whilst the information on the likelihood of a pulmonary nodule being detected on screening was informed by retrospective data on prevalence of pulmonary nodules in Hodgkin lymphoma survivors undergoing chest CT [30]. The Centre for Disease Communication ‘Everyday Words for Healthcare Communication’ booklet and online clear communication index tool guided the language used [31, 32].

### Input of the steering group

**Fig. 2:**
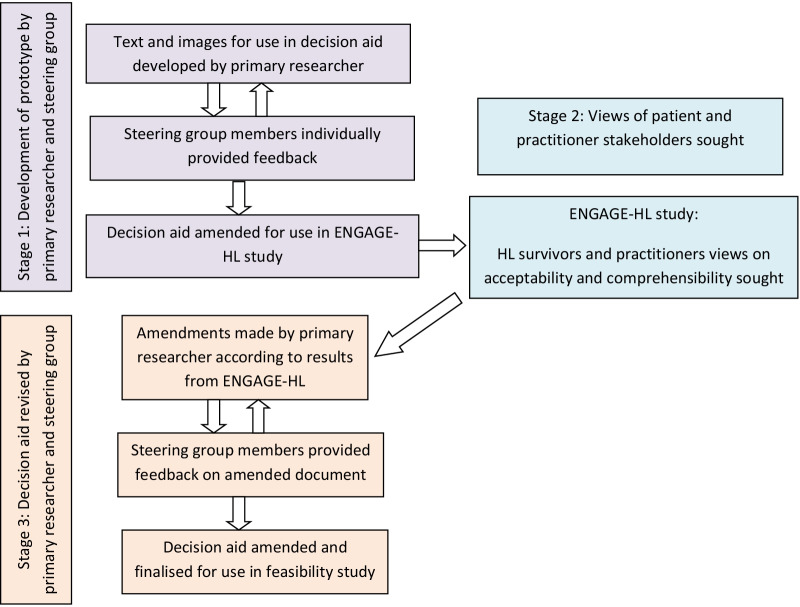
Flow chart of the decision aid development process

A steering group of clinical experts and patients was set up, comprising 9 individuals with expertise in lymphoma late effects, lung cancer screening, risk and cancer communication and 3 survivors of HL (2 female, 1 male). Between November and December 2020, all members of the steering group provided feedback on an initial prototype draft—developed by RB—which was subsequently revised to produce the version for further review by stakeholders. The steering group later commented on RB’s proposed amendments to the decision aid following review by stakeholders in the ENGAGE-HL study. The amendments made to the decision aid after the ENGAGE-HL study are described in Additional file 1: Table S3. A flow chart demonstrating the decision aid development process is shown in Fig. 2.

### The decision aid prototype

The decision aid prototype is a 16-page booklet, designed to be read in paper format, entitled *‘Screening to find the early signs of lung cancer after treatment for Hodgkin lymphoma: Helping you decide’*. The features of the decision aid prototype are detailed in Additional file 1: ‘Supplementary Information’: Table S1, but the decision aid is not publicly available at present.

**Table 1:** Personal characteristics of participants

	HL survivors		
	Online survey: participants personal characteristics n = 38	Focus group 1: n = 6	Focus group 2: n = 5
Gender	Female: 30	Female: 3	Female: 5
	Male: 8	Male: 3	
Median age (range)	44 (21–71)	(26–60)	(21–71)
Ethnicity	White British: 30	5 white British, 1 Spanish	All white British
	Other white background: 5 (1 Spanish, 1 Portuguese, 1 Polish, 1 not stated, 1 Irish)		
	Indian: 1		
	Pakistani: 1		
Smoking status	Never smoker: 25	Not captured	Not captured
	Ex-smoker: 12		
	Current smoker: 1		
Years since HL treatment:	< 5: 17	< 5 years: 4	< 5 years: 1
	5–10: 6	5–10 years: 2	5–10 years: 2
	11–15: 3		> 20 years: 2
	16–20: 1		
	> 20 years: 11		
Follow-up status	21 remain in follow-up	4/6 remain in follow-up	3/5 remain in follow-up
	17 discharged from follow-up		
Treatment for HL	Not captured	All received chemotherapy alone	Radiotherapy only: 1 Chemotherapy only: 2
			Both: 2
Level of education completed	Not captured	2: GCSE/O-level	2: A-levels/other college education
		1: A-levels	3: university educated
		3: university educated	

	Practitioners n = 11
Role (number)	Consultant haematologist (3)
	Senior registrar (doctor) (1)
	Advanced nurse practitioners (haematology/lymphoma) (3)
	Clinical nurse specialist in lymphoma (4)

### Testing the decision aid among stakeholders: the ENGAGE-HL study

A study using mixed quantitative and qualitative methodology was developed to assess the decision aid among people treated for HL and practitioners. Mixed methodology was chosen to facilitate quantitative analysis of the impact of the decision aid using validated scales, whilst qualitative methods were used to explore the perspectives of stakeholders in depth.

The specific study objectives were:

1. To assess the acceptability and comprehensibility of the decision aid amongst HL survivors and practitioners
2. To explore the decisional needs of HL survivors and informational and support preferences with regards to a lung cancer screening invitation
3. To explore the needs of practitioners providing support to survivors making the decision.
4. To assess the impact of the decision aid on HL survivors’ knowledge about lung cancer risk and lung cancer screening and on decisional conflict

### Theoretical framework

The Ottawa Decision Support Framework [33] describes the interaction of decisional needs, decision quality and decision support and asserts that unresolved needs negatively impact decision quality, which can adversely impact emotions, behaviour and health outcomes. Decision support strategies can improve decision quality by addressing unresolved needs, which may include inadequate knowledge and unrealistic expectations. This framework and the ‘Decisional Needs in Populations’ workbook [34] were used to develop the questionnaire items and topic guides for interviews and focus groups.

### Study design

There are two parts to the study. In Part A, HL survivors were recruited to take part in an online survey and /or a focus group whilst in Part B lymphoma practitioners were recruited to an interview study. Quantitative methodology was chosen to assess the decision aid’s impact on knowledge and decision making using validated scales and assessments, whilst the aim of the focus groups was to elicit the views of HL survivors’ by allowing participants to debate and to discuss their shared and diverse experiences. Interviews with practitioners were chosen for ease of scheduling and to avoid the potential for any practitioners feeling less able to share their perspectives due to having less experience or due to professional hierarchy.

Parts A and B of the study ran concurrently. HL survivors were eligible to participate if they were treated in the UK, were aged 18 or over and had not been diagnosed with lung cancer or participated in a lung cancer screening pilot. Practitioners were eligible if they worked in the UK in a clinical role treating or supporting HL patients.

### Recruitment

To recruit HL survivors to part A, a study advert was placed on the Lymphoma Action charity Twitter feed on multiple occasions over a 4 week period and posted twice on the Lymphoma Action Facebook support page. The study advert was also included in the Lymphoma Action magazine and in an email to Lymphoma Action members. The study advert directed interested individuals to contact the researchers or access the study website, which hosted the participant information sheet, researchers contact details, and a link to the online survey.

To recruit practitioners to Part B, a separate study advert was placed on the Lymphoma Action charity Twitter feed and further information was available on the Lymphoma Action website. Study details were also listed on the British Society for Haematology website. Practitioners were sent the participant information sheet by email. All participants (in both Part A and B) were offered a £30 e-voucher for their participation (if a HL survivor participated in the focus group and completed the online survey, they received two £30 vouchers). Recruitment to Parts A and B took place between March and July 2021.

### Part A study procedures

#### Online survey

Participants in part A completed an online consent form followed by a survey, hosted on the Qualtrics platform. The online survey captured demographic data and measured lung cancer screening knowledge and decisional conflict before and after the participant accessed a pdf version of the decision aid. Lung cancer screening knowledge was measured using a 16-item scale, adapted from a published scale [35], and decisional conflict was measured using the Low Literacy Decisional Conflict Scale (DCS) [36]. Additional novel questions explored aspects of decisional conflict, information and support preferences and acceptability of the decision aid. The Short Test of Functional Health Literacy Assessment [37] (S-TOFHLA) was administered at the end of the survey.

#### Focus groups

Two focus groups, lasting 60 min, took place using Zoom teleconferencing and were audio recorded. Participants were required to complete an online consent form and short questionnaire to collect personal characteristics prior to the focus group and were sent a pdf version of the decision aid to view at least 48 h prior to the focus group. The main researcher (RB) and a second moderator attended the focus groups.

### Part B study procedures

Semi-structured interviews, lasting 25–45 min, took place over telephone or Zoom and were audio-recorded. Practitioners completed an online consent form prior to the interview. A topic guide covered questions relating to the decisional needs of HL survivors considering lung cancer screening, the comprehensibility and acceptability of the decision aid and practitioners’ lung cancer screening informational needs.

### Data analysis

#### Online survey (part A)

Participant characteristics and their responses to questions exploring decisional conflict and support and information preferences are presented descriptively. To compare the difference in median total DCS scores and subscale scores and median proportion of correct answers given in the knowledge scale before and after exposure to the decision aid, the Wilcoxon signed rank test was used as the data did not meet tests for normality. McNemars test was used to compare screening intention before and after exposure to the decision aid. A significance level of 0.05 was used throughout. Effect sizes are presented using Cohens *d* values, defined as small (*d* = 0.2), medium (*d* = 0.5), and large (*d* = 0.8) [38]. The percentage of correct answers given to each question or statement in the knowledge scale and the median DCS scores and interquartile ranges are also presented descriptively.

#### Focus groups with patients (part A) and interviews with healthcare practitioners (part B)

The focus groups and interview recordings were transcribed intelligent verbatim.

Since the interview and focus group schedules covered similar topics, the framework method of content analysis was used [39] with the aim of identifying concordant and contrasting views among HL survivors and practitioners. NVivo 12 software was used to store and organise transcript files, codes and the framework matrix. The first author applied codes to the interview and focus group transcripts independently, producing two sets of codes, one for the interviews and one for the focus groups. This was an iterative process whereby codes were applied to the transcripts of the first seven interviews and the first focus group, and when the remaining interviews were transcribed and the second focus group had taken place, a further round of coding took place where previously developed codes, and new codes, were applied. Two researchers (RB and TS) met to discuss the codes and emerging themes, at which point any disagreements over coding were resolved. Subsequently, RB developed a coding framework which could be applied to both focus group and interview transcripts. During the development of the thematic analysis, the RB and TS met on multiple occasions to discuss the emerging themes. The results of the study have been made available on the study website (engagehl.com) but participants have not been involved in the analytic process.

#### Reflexivity statement

Focus group participants and practitioners were aware that the interviewer (RB) had been involved in developing the decision aid that they were reviewing. Being aware of this, the interviewer took care to facilitate a safe environment in which participants could openly express their views towards the decision aid—including negative ones—by encouraging participants to share both positive and negative views and seeking their views throughout of ways to improve it. The interviewer was willing to answer questions that participants had on the topic of lung cancer screening and to discuss the rationale behind decisions that had been made during the development of the decision aid.

During the second focus group, the researcher (RB) explored issues which had not been addressed in the first focus group or which merited further exploration and additionally explored issues that had emerged from an interim analysis of the online survey. Thus, the direction and structure of the second focus group was influenced by the preceding research activities. Although this was a useful opportunity to discuss certain survey findings, this meant there was less time during the second focus group for discussion which could have generated new perspectives.

During the coding and development of the themes, two researchers discussed the challenges associated with running the focus groups, including group dynamics. They acknowledged the potential for their experience of running the focus groups to influence the resulting codes and themes, and efforts were made to remain unbiased in the weight attributed to the views of individual participants when developing the thematic analysis.

## Results

### Part A

The online survey was completed by 38 HL survivors described in Table 1. In summary, the majority were female with a median age of 44, of white British ethnicity and were never smokers. There was a wide range of time since follow-up among participants and slightly more remained in follow-up than were discharged. All participants had adequate levels of health literacy according to S-TOFHLA.

#### Lung cancer risk and screening related knowledge

The median percentage of correct responses to knowledge questions and statements increased following exposure to the decision aid (68% pre exposure, 93% post exposure (*p* value < 0.001). The effect size was 1.4. The percentage of correct answers given to each question pre and post exposure to the decision aid is shown in the Additional file 1: ‘Supplementary information’: Table S2.

**Table 2:** Decisional conflict median scores pre and post exposure to the decision aid

	Pre: Median (range; interquartile range (IQR))	Post: Median (range; interquartile range) p value for difference in median pre and post scores	Effect size (Cohens d value)
Total DCS score	67.5 (0–100; IQR 40)	0 (0–80; 10) p < 0.001	1.9
Uncertainty subscale score	50 (0–100; IQR 80)	0 (0–100; IQR 6.25) p < 0.001	1.0
Informed subscale score	100 (0–100; IQR 37.51)	0 (0–66; IQR 0) p < 0.001	2.0
Values clarity subscale score	75 (0–100; IQR 56.25)	0 (0–100; IQR 0) p < 0.001	1.5
Support subscale score	33.33 (0–100; IQR 41.67)	0 (0–100; IQR 0) p < 0.001	0.7

#### Decisional conflict

In the decisional conflict scale and subscales, higher scores represent higher levels of decisional conflict, higher levels of uncertainty, feeling more uninformed, feeling more unsupported and feeling more unclear about personal values. Following exposure to the decision aid, median total DCS scores and median uncertainty, informed, values clarity and support subscale scores reduced indicating that the decision aid reduced levels of decisional conflict, reduced uncertainty, increased feeling of being informed, increased values clarity and feelings of being supported. Median scores, range, interquartile range, p-values for difference in pre-post median scores and effect size are shown in Table 2.

#### Intention to participate in a future lung cancer screening programme

Before and after accessing the decision aid, participants were asked: “If you were invited to go for a lung cancer screening test, would you go?” Prior to reading the decision aid, 33 (86.8%) participants responded ‘Yes, definitely’ and 5 (13.2%) responded ‘Yes, probably’. After reading the decision aid, 29 (76.3%) responded ‘Yes, definitely’ and 9 (23.7%) responded ‘Yes, probably’. The difference in strength of intention before and after reading the decision aid was not significant (p = 0.21).

#### Decision making and information and support preferences

Participants answered the following questions before accessing the decision aid. Responses to the question, ‘If you were invited to lung cancer screening, how easy would it be for you to make the decision?’ were as follows: ‘extremely easy’; 10 (26.3%), ‘quite easy’; 15 (39.5%), ‘neither easy nor difficult’: 10 (26.3%), ‘quite difficult’; 3 (7.9%). Participants rated their level of agreement to a series of questions assessing difficulties relating to decision making. The results are shown in Table 3.

**Table 3:** Responses to questions regarding difficulties in decision making

Statement	Response (n = 38) n (%)		
	Strongly disagree/disagree	Neither agree nor disagree	Strongly agree/agree
I would be unsure what to do	25 (65.8)	5 (13.2)	8 (21.0)
I would be worried what could go wrong	21 (55.3)	9 (23.7)	8 (21.0)
Trying to make the decision would upset me	31 (81.6)	3 (7.9)	4 (10.5)
I would be constantly thinking about the decision	26 (68.4)	5 (13.2)	7 (18.4)
I would delay making the decision	34 (89.5)	3 (7.9)	1 (2.6)

After reading the decision aid, 23 (60.6%) said they would not seek out more information, 13 (34%) would and 2 (5.3%) were unsure. Participants were asked to select the support options might be useful to them. Responses were as follows: searching the internet: 25 (65.8%), charity or organisation webpage: 29 (76.3%), talking to a doctor or specialist nurse: 28 (73.7%), asking a support group: 5 (13.1%).

Nineteen respondents (50%) said they would involve someone else in their decision making and all those responding this way indicated they would involve their family in the decision whilst 2 (5.3%) said they would involve their clinical team. When asked about the level of involvement of the doctor in decision making, 20 (52.6%) said they would decide on their own, 13 (34.2%) said they would decide after considering their doctor’s opinion, 4 (10.5%) would decide with their doctor, and 1 (2.6%) said their doctor would decide after considering their opinion.

#### Acceptability of the decision aid prototype

Thirty-three (86.8%) said the length was ‘just right’, whilst 5 (13.2%) said it was ‘too long’. Thirty-six (94.7%) participants said the amount of information in the decision aid was ‘just right’ whilst 2 (5.3%) said it was ‘too much’. Participants were asked to rate the way the information was presented within the different sections of the booklet. Their responses are shown in Table 4. Asked about the balance of the information, 28 (76.3%) said it was balanced, 9 (23.7%) said it was ‘slanted towards having a lung cancer screening test’ and 1 (2.6%) said it was ‘slanted towards not having a lung cancer screening test’. All participants said they would find the decision aid useful if they had to make a decision about undergoing lung cancer screening.

**Table 4:** Ratings given to sections of the decision aid

Section of the decision aid	Response (n = 38) n (%)	
	Excellent/good	Fair/poor
How likely is it that I will develop lung cancer?	31 (81.6)	7 (18.4)
What does lung cancer screening involve?	37 (97.4)	1 (2.6)
What are the benefits of having a lung cancer screening test?	37 (97.4)	1 (2.6)
What are the disadvantages of having a lung cancer screening test?	36 (94.7)	2 (5.3)
Making a decision	31 (81.6)	7 (18.4)
What are the symptoms of lung cancer?	34 (89.5)	4 (10.5)
Information and Support	36 (94.7)	2 (5.3)

### Thematic analysis of focus groups with HL survivors and interviews with practitioners

#### Focus group participant and practitioner characteristics

Whilst the first focus group was balanced in terms of gender, the second focus group was comprised of female participants only. The age range across both focus groups was 21–71 years of age. Most were of white British ethnicity. Of note, all the focus group participants had also completed the online survey. Practitioners were currently practicing as doctors or nurses. The nurses all held specialist roles (clinical nurse specialists or advanced nurse practitioners in the fields of haematology or lymphoma). Their characteristics are shown in Table 1.

### Theme 1: accessing and understanding the decision aid document

#### Acceptability

During the focus group, participants’ perspectives on the language, length and format were explored, with probing questions to generate a deeper understanding of viewpoints. All groups agreed that the language was clear and jargon-free, especially by focus group participants experienced in patient and public involvement and engagement (PPIE). The length of the decision aid (16 pages) was a cause for concern, however as information was felt to be “concise” and the layout “uncluttered” the length was generally considered manageable:

“Because it’s written so clearly and in such simple language once you start reading it it’s actually a lot quicker than you think” (Focus group 2 participant, female)

Linked to this, participants felt strongly that the decision aid document should be comprehensive despite its length and it was pointed out that recipients could “dip in and out of it”. Across the two focus groups, suggestions were made to improve the readability through simple format changes, such as the use of bullet points and bold headings. It was suggested that videos or forums may be a better source of information and support for patients less likely to read written information.

#### Comprehension of lung cancer risk information

During the focus groups, it emerged that participants had become aware of the treatment-related risk of lung cancer for the first time through participation in this study and had therefore not been previously exposed to data relating to this risk. This lack of prior awareness impacted their perceptions of the absolute risk values that were presented in the decision aid. Those who perceived the values to be lower than they had anticipated gleaned some reassurance, but this was not universal. A female focus group participant who was treated at a young age said she had not expected to get lung cancer, so the values still appeared “quite high”. In relation to this, it appeared that using icon arrays to support textual information on absolute risk aided comprehension and reduced affective risk perceptions.

“With regards to it being simple for others to read, I definitely found the graphics useful from that perspective just to get a real insight. You can say four in 100 people, but when you see it in an infographic it’s much more impressionable I guess, and you relax a bit more and your anxiety leaves, that actually the chances are that’s probably not me.” (Focus group 1 participant, female)

Practitioners also viewed the icon arrays positively, saying they were a simple but effective method of communicating risk.

Practitioners felt it was important for recipients of the decision aid to be able to identify the treatment related risk factors relevant to them. In keeping with this, there were multiple occasions when focus group participants correctly identified their personal risk factors using the information in the decision aid. When participants could not see their chemotherapy regimen listed as a risk factor, they sought clarification from the researcher running the focus group.

Whilst the decision aid provided information on risk factors and the absolute risks relating to single and combined modality treatments, it was not tailored to individual recipients. One participant expressed concerns about this, saying that the information was not sufficient for her to understand her personal risk factors.

“I think it certainly doesn’t answer all the questions that I would have as to why I would be at risk personally. But you’re never going to cover that off, that’s the problem, in a leaflet. So, I think it does a good job of being quite generic and covering off the main reasons, without being specific; you’d have to reach out elsewhere.” (Focus group 1 participant, female)

Both practitioners and focus group participants raised concerns that the inclusion of lifetime cumulative lung cancer risk values for the general population (7–8/100) was confusing. They felt that these data contradicted the text which stated that HL survivors were at higher risk than the average person, because the lung cancer absolute risk value for HL survivors 35 years after treatment was 4–5/100, seemingly less than the general population. Practitioners widely recommended that alternative data be used.

#### Facilitating informed decision making

Across both focus groups, participants felt that the decision aid presented lung cancer screening as a choice rather than a recommendation. It was widely agreed among practitioners and focus group participants that presenting pros and cons in textual and summary table format would help facilitate informed decision making by helping people identify the issues that were most salient to them. Being able to weigh up pros and cons during decision making held more importance for some focus group participants than others, for example one participant who perceived a prior lack of involvement in decision making relating to her cancer diagnosis, said:

“I think that’s so important, especially when some of those decisions are taken completely out of your hands when you’re diagnosed with cancer.” (Focus group 1 participant, female)

In contrast, another participant indicated that the risks associated with screening were of minimal importance to them if there was any potential benefit: “If I know it’s going to help or it’s going to try and help us I’ll just do it.” (Focus group 1 participant, male).

Participants in the second focus group were asked to consider whether the decision aid was slanted towards lung cancer screening, which had been reported in the online survey analysis. There was agreement among them that the document was balanced and that the pros and cons of screening were described in equal detail. One participant wondered whether it was biased to present pros before cons but felt this was the “right decision” as presenting cons first may dissuade people from reading about the potential benefits.

### Theme 2: factors influencing lung cancer screening participation decisions

#### Perceptions of radiation risk associated with lung cancer screening

Participants were asked to consider the amount of information contained in the decision aid on the radiation risk associated with lung cancer screening. In the ensuing discussion, it emerged that the extent to which focus group participants were concerned about the radiation risk associated with screening was variable. In discussing this, two male participants agreed that although radiation could have adverse consequences, this knowledge would not prevent them from accepting a lung cancer screening test due to the potential benefits associated with early detection.

Another male participant said that whilst he placed more importance on radiation risk now that he was in remission, it remained a minor concern in view of previous cancer treatment:

“I guess from a fact point of view you can bombard me with anything else. You sign a form and bags of stuff arrive that say deadly on them with a skull and cross bone.” (Focus group 1 participant, male)

Conversely, one female participant said that as a young adult, her level of concern about radiation risk would be greater if regular screening was recommended over a long time period, whereas she would not be concerned about a single scan. For another female participant, the differing impact of radiation on men and women was an important consideration, for example in relation to fertility. In general, practitioners perceived that radiation risk would be a minor concern for patients in view of having undergone multiple scans*:*

“When you think of all the scans our patients have, it’s nothing really, is it?” (Clinical nurse practitioner)

#### A screening offer can provide a degree of reassurance about lung cancer risk

Health-related anxieties experienced by HL survivors, particularly regarding cancer recurrence but also about developing late effects of treatment, were discussed by both focus group participants and practitioners. Practitioners felt that anxiety and “hypervigilance” about their health would lead most survivors to take up an offer of lung cancer screening, making the decision a straightforward one.

“I think some people would bite your hand off to go and reassure themselves there’s nothing wrong” (Advanced nurse practitioner)

Additionally, practitioners felt that although an offer of lung cancer screening could exacerbate anxiety, survivors could be reassured by a screening offer. In considering this, they cited their experience of patients’ enthusiasm for surveillance imaging during follow-up. Focus group participants and practitioners went on to discuss the delivery of information about lung cancer risk. Both groups felt that delivering information about lung cancer risk in the context of an invitation to screening—accompanied by an explanation about the rationale—might somewhat mitigate the anxiety associated with becoming aware of this risk, although it was also said that reassurance could be short lived if regular screening were not available. Both groups noted that information on risk of late effects was often given without an offer of surveillance or screening.

“I’d find this arriving kind of reassuring cause it means someone’s actually monitoring, checking up on you and not just leaving you to your own devices afterwards so you guys have assessed the risk and doing something about it which we don’t get very much to be honest its more just, ‘oh there’s a risk’ and they leave us alone.” (Focus group 2 participant, female)

#### Patient age at approach about lung cancer screening

Practitioners were asked about the challenges survivors might face when considering undergoing lung cancer screening. The age at which patients were approached about lung cancer screening was felt to be an important consideration. Practitioners felt that younger patients’ desire to “move on” from their illness might render them less likely to engage with information about late effects and screening. In contrast, they felt that people contacted about lung cancer screening at an older age would have better “emotional capacity” to understand late effects information and engage with screening because they or their peers may be experiencing health problems, making health a more salient issue and higher priority. In contrast with this, a focus group participant who was treated in their sixties and currently aged over 70 said that being treated at an older age led them to feel less concerned about lung cancer as a late effect, as they were not sure they would live long enough to be affected. Although the desire to avoid “remedicalisation” could reduce engagement with screening in all age groups, practitioners thought this may be particularly relevant to people diagnosed at a young age:

“I think there will be some who will have a real issue with that identity of being someone who’s still…who can possibly still get ill from something serious again in the future” (Lymphoma doctor)

### Theme 3: information provision and support

#### Lung cancer screening discussions: past and future practice

There was a perception among practitioners that although late effects had not been widely discussed with patients in the past, this had improved in recent years. Nevertheless, there was evidence of variation in current follow-up strategies and timing of discussions about late effects and screening opportunities, which one practitioner attributed to a lack of guidance.

“I don’t think we have clear enough guidance that we can use uniformly across our Hodgkin lymphoma survivors and that’s tailored to each patient as well.” (Consultant haematologist)

This was reflected in the focus groups, where participants described varied experiences of follow-up care and management of late effects. Participants appeared uncertain about how to access support around late effects and the one participant who had accessed a late effects clinic had done it through “self-advocacy”. Practitioners felt that if lung cancer screening were to become established in future, HL patients should be “forewarned” about future screening invitations whilst still in follow-up to mitigate the shock they might experience on receiving an invitation years later. Although practitioners did not offer a consensus as to the optimal time to deliver lung cancer screening information during the follow-up period, some perceived that patients would not be receptive to this screening information until they had achieved remission, as they would be focussed on getting through treatment.

“Screening would be something I would definitely want to talk about at the end of treatment rather than right at the beginning when they’ve already got those additional stresses.” (Consultant haematologist)

#### Sources of information and support for HL survivors and their practitioners

When discussing support and information, practitioners anticipated that HL survivors would prefer to access support and advice through their own clinical team with whom they had established a relationship and would be likely to follow the recommendation of their lymphoma physician, whose view they would “trust”. Indeed, focus group participants expressed their desire to seek advice from their clinical team and it appeared that a positive screening recommendation could be influential.

“If my consultant says to me take it, okay I’ll be there in five minutes, that’s my attitude.” (FG1, male).

Practitioners acknowledged that patients long discharged from follow-up may not have an obvious point of contact, in which case they might seek support from a variety of other sources including a designated nurse specialist for their local area, their GP, or patient charities. Family members were considered to be important and influential sources of support during decision-making.

Practitioners were asked how they might be informed and supported should lung cancer screening become available for their patients in future. Clinical nurse specialists said that having access to the same decision aid document given to patients would fulfil their informational needs, whilst some doctors felt more detail on risk stratification would be useful for them to discuss risk with patients. Nurse specialists did not anticipate difficulties in providing psychological support to patients, saying that this was a key part of their role.

## Discussion

This paper describes our approach to developing a decision aid and shows that the decision aid significantly improved lung cancer risk and screening related knowledge and reduced decisional conflict among HL survivors. Although the decision aid improved participants’ knowledge on treatment related lung cancer risk factors, the degree of improvement varied. For example, most participants were already aware of the lung cancer risk associated with radiotherapy, but far fewer had prior knowledge of the risk associated with chemotherapy. This may reflect the nature of information previously provided to participants about lung and other second cancer risks associated with radiotherapy. Nevertheless, it can be argued that even modest improvements in knowledge are of value because an improvement in knowledge around options and outcomes improves decision quality [40]. Decisional conflict scores reduced after accessing the decision aid across all subscales but the smallest change in pre-post median scores and the smallest effect size was seen in the ‘support sub-scale’, possibly reflecting the fact that more than half of participants remained in follow-up, retaining access to their clinical team for advice.

Participants in the online survey universally expressed willingness to undergo lung cancer screening if invited, even before accessing the decision aid. The utility of a decision aid in a population who are already highly swayed towards one option—screening—could be questioned, but it can be argued that recipients who strongly favour an option at the outset would benefit from feeling better informed, supported and clearer in their values, thus improving the quality of the decision-making process and quality of the choice made [40, 41]. Notably, after accessing the decision aid, a higher proportion responded that they would ‘probably’ attend lung cancer screening, as opposed to ‘definitely’. The improvement in decisional conflict scores after viewing the decision aid would suggest that this change in strength of intention could be a result of participants being more informed of their level of lung cancer risk and of the risks of screening, as opposed to feeling less certain about the decision they would make. Although not statistically significant, this finding highlights that becoming more informed can move screening intentions in both directions. This was demonstrated in the aforementioned Cochrane review of decision aids for people facing screening decisions, where there were mixed results in terms of uptake of breast and colorectal cancer screening after exposure to a decision aid [3].

Our approach to the development of the decision aid—a schematic outline is shown in Figure 2-diverged from the systematic approach recommended by Coulter et al*.* [42]. Since the decision aid development took place as part of RB’s doctoral research, the timeframe for development of the prototype was short and for this reason RB designed the prototype for review by the steering group. In addition, patients’ needs relating to lung cancer screening decision making were not assessed prior to developing the decision aid prototype. Prior qualitative research exploring the perspectives of HL survivors on lung cancer screening showed that most survivors were unaware of lung cancer risk [17] and since there is no lung cancer screening programme for HL survivors, we anticipated that lung cancer screening related knowledge would be minimal in this group. We therefore opted to develop a prototype based on the comprehensive IPDASi and then explore the extent to which it met patients’ needs, with the intention of amending the DA accordingly prior to further use.

### Strengths and limitations

A particular strength of this work was that patient and practitioner stakeholders were involved at every level of the development process. Although our approach did not fully reflect the recommended systematic approach described by Coulter et al., stakeholder feedback influenced the decision aid design in that amendments were made following initial feedback from the steering group and then again—with the input of the steering group—taking into account the results of the ENGAGE-HL study.

The use of mixed methodology allowed us to quantify the impact of the decision aid through validated scales and to explore the perspectives of stakeholders and the issues pertinent to patients when facing lung cancer screening decision through qualitative methods. In addition, the use of mixed methodology led to specific insights. For example, the lack of personally tailored information caused ongoing decisional conflict for some focus group participants despite the online survey demonstrating a significant reduction in decisional conflict. The framework method of analysis allowed us to identify areas of concordance and discordance between patients and practitioners. For transparency and rigour, we have reported the evaluation of the decision aid according to the Standards for UNiversal reporting of patient Decision Aid Evaluation (SUNDAE) checklist [43] although not all checklist items were relevant in this early phase of evaluation.

A limitation of this study was the convenience method of sampling of HL survivors, which in turn limits the extent to which the decision aid can be considered acceptable and comprehensible to the wider population of HL survivors. We did not stipulate that patient participants received treatments that increased their lung cancer risk since this would require accurate recall of chemotherapy drugs and radiation site. Therefore, some participants were not at excess risk of lung cancer, meaning not all participants were representative of the intended target group for the decision aid. Nevertheless, a majority were considered to be at excess risk because of treatment trends in the last 40 years—this was the case for the focus groups where participants volunteered their treatment details—and the views of those who were not remain pertinent and relevant to our research questions. A further limitation of the convenience sampling strategy was that men, current smokers and non-white ethnicities were poorly represented among the patient participants meaning decisional needs unique to these groups would not have been identified. Health literacy levels were high among survey participants and more than half of focus group participants were university educated. Given that 42% of working-age adults in the UK cannot understand everyday healthcare information, rising to 61% when numeracy is required for comprehension [44], recipients of the decision aid within a future study may be less able to understand the decision aid than our participants. All participants accessed the decision aid in a digital pdf format due to the research being conducted virtually. However, in the future study, the decision aid will be in the form of a paper booklet. Recipients of the paper booklet may have different views regarding its’ acceptability than our participants who accessed it online.

### Conclusion

The findings of this study suggest that the decision aid developed here would support informed decision making when provided to HL survivors considering undergoing lung cancer screening. Its’ suitability for use in a larger population who are more diverse in terms of ethnicity and educational level is uncertain and further research is warranted in those groups. Informed by the results of this study, the decision aid prototype tested here has been developed further by the steering group to produce a decision aid document which will be used in a future feasibility study of lung cancer screening in HL patients [45]. In this future study, the impact of the decision aid on knowledge, decisional conflict and preparedness for decision making will be tested in a larger sample.

## Supplementary Information

**Additional file 1.** Supplementary Information.

## Data Availability

All data generated or analysed during this study are included in this published article and its’ Additional file 1.

## Abbreviations

DA: Decision aid
HL: Hodgkin lymphoma
LCS: Lung cancer screening
IPDASi: International Patient Decision Aid Standards instrument
DCS: Decisional conflict scale
S-TOFHLA: Short Test of Functional Health Literacy Assessment
SUNDAE: Standards for UNiversal reporting of patient Decision Aid Evaluation
