# Supplementary material for: The development of a decision aid to support Hodgkin lymphoma survivors considering lung cancer screening
Source: BMC Med Inform Decis Mak. 2022 Feb 1;22:29. doi: 10.1186/s12911-022-01768-y (PMC8805261; doi:10.1186/s12911-022-01768-y)
Supplement: Supplementary file 1 — Additional file 1. Supplementary Information. [file 12911_2022_1768_MOESM1_ESM.docx]

Table 1: Features of the decision aid prototype

| Page | Content |
| --- | --- |
| Title page | ‘Screening to find the early signs of lung cancer after treatment for Hodgkin lymphoma: Helping you decide’, followed by ‘A lung scan can detect the early signs of lung cancer before symptoms have developed’.  Cartoon of magnifying glass held over a lung |
| Page 2 | Contents page |
| Pages 3-5 | Text detailing risk factors for lung cancer following treatment for Hodgkin lymphoma    Text and icon arrays describing:   1. The 35-year post treatment cumulative incidence in men and women 2. The absolute risks of developing lung cancer after chemotherapy alone or chemotherapy and radiotherapy combined. Data on the lifetime incidence of lung cancer in the general population is provided.     Data on the lifetime incidence of lung cancer in the general population |
| Page 6 | Text describing the low-dose CT scan procedure accompanied by image of person going through CT scanner  Text detailing process for getting the results |
| Page 7 | Text and icon array describing likelihood of negative (clear) result or needing extra tests to rule out lung cancer |
| Page 8 | Text (2-3 sentences) addressing the following questions:  What happens if the scan is clear?  What happens if the scan result is uncertain?  What happens if a possible lung cancer is seen?  What else might the scan show?  How often can I have a lung scan? |
| Pages 9-11 | Text risks and benefits of undergoing lung cancer screening |
| Page 12 | Common symptoms of lung cancer |
| Pages 13-14 | Header: Making a decision  Pros and cons table  Suggested steps to help decision making |
| Page 15 | Information and support sources |
| Page 16 | Text boxes to write down pros and cons ‘that are important to you’ and any questions about lung cancer screening |

Table 2: The proportion of correct responses to the knowledge scale pre and post exposure to the decision aid

| Question / statement  *(response options with correct answer in bold)* | Correct responses (%) | |
| --- | --- | --- |
|  | Pre exposure | Post exposure |
| A lung cancer screening scan will spot cancers 100% of the time  *(Yes/****No****/I don’t know)* | 50 | 97 |
| Most spots on the lung seen on a screening scan are cancerous  *(Yes/****No****/I don’t know)* | 57 | 94 |
| If a lung cancer screening test is clear (cancer is NOT found), you won’t develop lung cancer in the future  *(Yes/****No****/I don’t know)* | 84 | 100 |
| Lung cancer found on a screening scan can always be cured  *(Yes/****No****/I don’t know)* | 84 | 97 |
| A lung cancer screening scan can tell you if you are likely to develop lung cancer in the future *(True/****False****/Unsure)* | 23 | 65 |
| How many people with an abnormal CT scan will have lung cancer? *(Most will have lung cancer / About half will have lung cancer /* ***Most will not have lung cancer*** */ I don’t know)* | 23 | 84 |
| Can a CT scan miss a tumour in your lungs? *(****Yes****/No/I don’t know)* | 44 | 76 |
| Will all tumours found in the lungs grow to be life-threatening? *(Yes/****No****/I don’t know)* | 55 | 97 |
| Without screening is lung cancer often found at a late stage when cure is less likely? *(****Yes****/No/I don’t know)* | 73 | 100 |
| How much does screening for lung cancer with a CT lower your chance of dying of lung cancer? *(About 95% / About 50%* ***/ About 20%*** */ I don’t know)* | 10 | 57 |
| Can a CT scan find problems other than lung cancer? *(****Yes****/No/I don’t know)* | 97 | 100 |
| Is radiation exposure one of the harms from lung cancer screening? *(****Yes****/No/I don’t know)* | 68 | 89 |
| Can radiotherapy to your chest increase your risk of getting lung cancer? *(****Yes****/No/I don’t know)* | 89 | 97 |
| Can chemotherapy increase your risk of getting lung cancer? *(****Yes****/No/I don’t know)* | 57 | 100 |
| Are you still at risk of getting lung cancer if you have stopped smoking? *(****Yes****/No/I don’t know)* | 94 | 100 |
| Can people treated for Hodgkin lymphoma who have never smoked get lung cancer? *(****Yes****/No/I don’t know)* | 92 | 100 |

Table 3: Changes made to the decision aid as a result of the ENGAGE-HL study

| Section of the booklet | Change made to the decision aid |
| --- | --- |
| ‘Why should I think about lung cancer screening?’ (page 2) | Addition: You have been identified as someone who was given one or more of the treatments that increase the risk of lung cancer.    Rationale: Patients may not recall their treatment or may think they are expected to determine their risk themselves |
| ‘Which treatments increase the risk of getting lung cancer? (page 2) | Addition: There is no evidence that ABVD increases the risk of lung cancer    Rationale: Patients who received this commonly used regime, but are at risk because of another regimen of chest radiotherapy, may wish to know whether ABVD also increases risk |
| Page 4 | The graphic showing risk in men and women has been combined into 1 chart    Rationale: to ensure length not increased by other additions |
| Page 4-5 | Addition:    Your chance of developing lung cancer depends on:    1. Whether you have ever smoked    · The chance of getting lung cancer is much greater in people who have smoked at any time.  · Most people who get lung cancer after Hodgkin lymphoma have smoked.”    Rationale: |
| Page 5 | Addition:  Other things to know  The risk of lung cancer in people treated for Hodgkin lymphoma is around 5 times higher than people in the general population who were not treated for lymphoma    Rationale: providing lifetime incidence rates for the general population led to confusion over the excess risk in HL survivors |
| Page 9 | Addition:  Can I have more lung screening scans after this study ends?    · No. At the moment, lung cancer screening is not routinely available outside of this study.  · If lung cancer screening does become available for you in the future, you will be contacted.    Rationale: after participating in the study, HL survivors would want to know if they could access further screening |
| What are the common symptoms of lung cancer? (page 12) | Addition:  Occasionally, people treated for Hodgkin lymphoma have some these symptoms because of their cancer treatment, sometimes for many years after treatment. For them, some of these symptoms are ‘normal’.    However, if you experience these symptoms and they are **not** normal for you, or if your usual symptoms **change**, it is important for you to speak to your GP.    Rationale: HL survivors can experience long-term respiratory symptoms after treatment, some of which are also symptoms of lung cancer |
| Pros and cons of lung cancer table (page 13) | The statement “You are less likely to die of lung cancer” was removed    Rationale: Lack of evidence for this statement in this population |
| Page 16: More information and support | Addition:  To be directed to the journal articles containing the data used in this booklet, please email…    Please note, the articles are written in scientific language which may be difficult to understand.    Rationale: Some survivors may wish to read the evidence behind the information in the booklet themselves |
| Worksheet (last page) | Separate text boxes removed so worksheet is blank    Rationale: more flexibility |
